# One-Stage Anchor-Free 3D Vehicle Detection from LiDAR Sensors

**DOI:** 10.3390/s21082651

**Published:** 2021-04-09

**Authors:** Hao Li, Sanyuan Zhao, Wenjun Zhao, Libin Zhang, Jianbing Shen

**Affiliations:** 1 Beijing Laboratory of Intelligent Information Technology, School of Computer Science, Beijing Institute of Technology, Beijing 100081, China; lih627@bit.edu.cn (H.L.); shenjianbing@bit.edu.cn (J.S.); 2 State Key Laboratory of Smart Manufacturing for Special Vehicles and Transmission System, Inner Mongolia No.2 Mailbox, Baotou City 014030, China; zhaowenjun9930@126.com (W.Z.); Z18686168230@163.com (L.Z.)

**Keywords:** 3D detection, anchor-free detector, one-stage detector

## Abstract

Recent one-stage 3D detection methods generate anchor boxes with various sizes and orientations in the ground plane, then determine whether these anchor boxes contain any region of interest and adjust the edges of them for accurate object bounding boxes. The anchor-based algorithm calculates the classification and regression label for each anchor box during the training process, which is inefficient and complicated. We propose a one-stage, anchor-free 3D vehicle detection algorithm based on LiDAR point clouds. The object position is encoded as a set of keypoints in the bird’s-eye view (BEV) of point clouds. We apply the voxel/pillar feature extractor and convolutional blocks to map an unstructured point cloud to a single-channel 2D heatmap. The vehicle’s Z-axis position, dimension, and orientation angle are regressed as additional attributes of the keypoints. Our method combines SmoothL1 loss and IoU (Intersection over Union) loss, and we apply (cosθ,sinθ) as angle regression labels, which achieve high average orientation similarity (AOS) without any direction classification tricks. During the target assignment and bounding box decoding process, our framework completely avoids any calculations related to anchor boxes. Our framework is end-to-end training and stands at the same performance level as the other one-stage anchor-based detectors.

## 1. Introduction

Object detection is one of the basic but challenging tasks in the field of computer vision. It promotes the development of a series of other tasks, such as instance segmentation and person re-identification and tracking. The task of 3D detection is to detect the 3D information of an object in the world coordinate system, including the position, size and orientation of the object. To get accurate 3D information, additional sensor data, such as RGB-D images or LiDAR point clouds, are used as input access object detection framework. Recent methods such as RoarNet [1] and Frustum ConvNet [2] first detect the 2D bounding box of the vehicle in the image, then extract the point cloud features in the bounding box through projection relationship to obtain 3D information of the object. Other methods, such as VoxelNet [3], SECOND [4] and PointPillars [5], are based on LiDAR point clouds and implement detection algorithms from a set of 3D bounding boxes on the ground plane. Of the two categories of method, the former focuses on multi-sensor information fusion, while the latter focuses on analyzing and processing point cloud information. There are two key differences between images and LiDAR points: (1) The 3D object projected into a monocular image will lose a dimension, which will cause scale variety, occlusion and deformation. The shape of the object on the image varies according to the viewing direction. It is difficult to recover 3D information from a monocular RGB image. However, in the LiDAR coordinate system, since the specific (x,y,z) for each point is known, there is no scale diversity problem for the same object. Generally, LiDAR is deployed from above the autonomous vehicle. There is less of an occlusion problem from the bird’s-eye view (BEV). A neural network based on the point cloud can infer the 3D information of the vehicle through the partial LiDAR-points information of the vehicle [3,6]. Since vehicle 2D detection results will not overlap from a BEV, it is helpful for the design of the training label. (2) The image is stored in a densely structured format. Experience has shown that traditional 2D convolution is suitable for processing such data. LiDAR points are non-uniformly sampled over the entire 3D space. An unstructured and unordered data format stores the point cloud, which cannot be processed directly by convolutional networks.

However, whether the above detection algorithms are 2D pre-detection or 3D detection based on point clouds, a series of anchor boxes need to be predefined. The anchor box is generally the average size of the same kind of object and has a fixed interval for the orientation angle. The output of the detection algorithm is tightly coupled to the parameters of the anchor boxes. Hence, for different kinds of objects or different data sources, these methods have to redesign the anchor boxes and fine-tune the output of the network, which is cumbersome and inefficient. Moreover, due to the anchor boxes, it is necessary to assign a label to each anchor box during the training process, which causes a large computational burden. For instance, given a point cloud corresponding to the horizontal 80 m × 60 m, the algorithm usually generates feature maps with the size of 400×300 and computes the similarity between 240,000 anchor boxes and about 3 to 10 ground truth 3D boxes.

In recent years, the anchor-free 2D detectors have developed rapidly. Anchor-free methods model the detection problem as keypoints matching or image segmentation, which is different from the anchor-based method. For example, the anchor-based methods employ multi-class dense classification to avoid occlusion problems. On the contrary, the anchor-free methods, such as FCOS [7], perform complex judgment logic for each pixel in the training process to assign a correct category label. There are currently artificial deep neural networks, such as PointNet [8] and SqueezeSeg [9], that are capable of extracting point cloud features and structured storage. In that way, we can learn a fixed size feature map from the point cloud, and draw on the ideas of 2D detection methods.

In this paper, we we propose an anchor-free, one-stage 3D detector. Our method focuses on detecting the other vehicles’ 3D information from the point clouds. We redesigned the target assignment and inference process, which completely avoids complex calculations involving anchor boxes. The network structure contains three modules, i.e., a point cloud feature extractor, the backbone network and the anchor-free detection head, which are tightly coupled. Since the vehicles are rigid and distributed on the ground plane, we detect objects from a BEV, which mitigates the adverse effects of occlusion and deformation. We firstly group LiDAR points from the raw point cloud, and extract their features with a parameterless voxel feature extractor or pillar feature extractor. The features are stored into a structured feature map. Then we apply fully convolutional networks in order to generate the heatmap and other regression parameters. The heatmap is encoded by the center points of the objects. Pixels with higher response values in the heatmap are more likely to be the target objects. The absolute offsets, Z-axis information, size and rotation angle of the object are calculated in regression branches, which are in parallel to the heatmap branch. We combine the SmoothL1 loss and IoU loss for training and redesigning the labels for regression. Compared with other methods, our contributions are listed as follows:

- We apply (cosθ,sinθ) for rotation regression as PIXOR [10]. Without any pivot angle prior and auxiliary angle classification branches in anchor-based methods, the unique orientation angle can be decoded by the result of our regression branch and achieve high average orientation similarity results.
- We combine SmoothL1 and high-level IoU loss for training. The SmoothL1 loss trains each regression branch separately, while IoU loss uniformly trains all regression parameters. The experimental results show that our detector achieves nearly the same performance level as the other anchor-based detectors. We also analyze the performance between the anchor-based and the anchor-free methods.

## 2. Related Work

3D object detection. The Mono3D [11], Deep3DBox [12] and 3DOP [13] focus on the front view RGB imagery, but the front view-based methods are easily susceptible to occlusion, which leads to missed detection. LiDAR point clouds provide more accurate position information than front view-based RGB images. Due to the sparse and unstructured data format of the LiDAR point clouds, learning features from point clouds is still a research hotspot. PointNet [8] uses a shared multi-layer perceptron to extract the features of each LiDAR point. PointNet++ [14] selects LiDAR points by the farthest point sampling method, and then uses ball center querying based on the selected LiDAR points to get the grouped point clouds. Finally, the classification and segmentation results were computed by PointNet layers. The current 3D detection methods are based on the point cloud learning algorithm from a bird’s eye view. The MV3D [15] encoded the sparse 3D point clouds with a compact, multi-view, hand-crafted feature representation for 3D proposal generation. Zhou and Tuzel [3] proposed VoxelNet for 3D object detection. It groups LiDAR points with voxels for 3D object detection. However, the voxel feature extractor suffers from a high computational burden because of the fully connected network and the cascading operation. The SECOND [4] applied sparse convolutional operation for the LiDAR point cloud feature extraction, to improve the speed of inference. Lang et al. [5] introduced a pillar-based 3D encoder, which converted a point cloud to a sparse pseudo image. Then, they removed the 3D convolutional module and processed the pseudo-image to high-level representation merely with 2D convolutional blocks. The PointPainting [16] was an effective sequential fusion method, which used a semantic segmentation network prediction from RGB images to enhance the point cloud features. These one-stage 3D detection heads adopted a set of predefined 3D anchor boxes. Jason et al. [17] proposed an aggregate view object detection architecture. They proposed the RGB image feature and BEV point cloud feature fusion method and geometric constraints to the bounding box regression process. It was cumbersome to calculate the label for each anchor box during training and pre-processing. There are many two-stage 3D detectors. Inspired by Faster R-CNN [18], Shi et al. [19] proposed a two-stage 3D detection method based on point clouds, PointRCNN. It first achieved foreground and background segmentation from point clouds by PointNet++ [14]. Then for each foreground point, it predicted a 3D object. The second-stage sub-network refined the proposals and generated the final 3D bounding boxes. F-PointNet [20] applies a 2D-driven 3D object detection method. It generates the 2D region proposal on the RGB-D image through Mask R-CNN [21], and combines the depth of the region to get the frustum proposals. The LiDAR points in a frustum proposal are used to generate the instance segmentation and 3D bounding boxes by PointNet++ [14]. PI-RCNN [22] uses a point-based attentive contfuse module to fuse features from multiple sensors. Pseudo-LiDAR++ [23] uses stereo camera images with LiDAR points to generate dense pseudo point clouds and enhance the performance of 3D detectors. Two-stage 3D detectors still cannot be trained end-to-end. AFDet [24] and CenterNet3D [25] were 3D anchor-free detectors that treated objects as keypoints for detection. AFDet [24] used a Euclidean distance transform for target assignment and a multi-bin method for orientation regression. CenterNet3D [26] was trained through an auxiliary corner attention branch and balanced L1 loss to improve average precision (AP).

Anchor-free detectors. The anchor-free algorithms took advantage of the methodology in related tasks of computer vision, such as segmentation [27,28,29] and pose estimation [30,31]. A famous anchor-free detector, YOLOv1 [32], predicts categories for each cell on the RGB image and regresses the bounding boxes. DenseBox [33] crops training samples from the raw image. The positive classification label of a target is encoded as a filled circle in the center of the bounding box. FCOS [7] classifies each pixel in the object’s bounding box and regresses the distance between the pixel location and the bounding box, which produces intensive prediction results for a target. FoevaBox [34] proposes the object fovea for the target assignment. All these methods effectuate approximately the same performance as anchor-based methods in a segmentation-like way. CornerNet [35] and CenterNet [26] use keypoint estimation for object detection. They got the heatmap of keypoints from fully convolutional networks and regressed the object size information as an additional attribute of the keypoints. The main difference between anchor-based and anchor-free methods is how to define the samples with corresponding ground truth labels during the training process.

## 3. Our Approach

**Figure 1:**
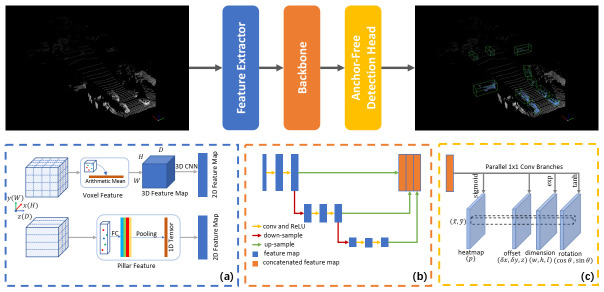
Our network architecture contains three modules. (**a**) We generate a 2D point cloud feature map with the pillar/voxel feature extractor. (**b**) Our backbone network consists of convolutional and deconvolutional blocks to extract multi-level semantic information. Note that the down-sampling factor can be one in application. (**c**) Our anchor-free detection head obtains the heatmap and absolute 3D additional information of the target through the fully convolutional network.

Our method consists of three modules, i.e., the point cloud feature extractor, the backbone network and the anchor-free detection head. Figure 1 shows our network architecture. We introduce our method in the following subsections. In Section 3.1, we generate the structured feature map in the bird’s-eye view from the point clouds. Then, the backbone network for processing point cloud feature map is described in Section 3.2. We explain the specially designed anchor-free detector in Section 3.3.

### 3.1. Point Cloud Feature Extractor

The point cloud data form an $N_{p}\times 4$ matrix which contains $N_{p}$ points with 3D locations (x,y,z) and reflection *r*. According to the spatial information, the point cloud is divided into a fixed number of groups by clustering. Then all the LiDAR points in each group are mapped into a feature vector in the same dimensions. In that way, we generate a structured representation of the point cloud. To build a structured 2D feature map from the point cloud, we test two types of point cloud feature extractors, the pillar feature extractor and the voxel feature extractor, as depicted in Section 3.1.2 and Section 3.1.3.

#### 3.1.1. Viewpoint Selection

Our goal is to format the point cloud into a 2D feature map similar to the image array. Reasonable selection of viewpoint is helpful for subsequent processing. There are two viewpoints regarding the formatting of the point cloud data. One is to generate a front-view point cloud array through spherical projection [9] or parallel projection, and the other is to process the point cloud directly from a bird’s-eye view. The spherical projection generates a front-view image-like data structure from the point cloud. In SqueezeSeg [9], the front view area of $90^{\circ }$ is divided into 512 grids. The pixel distance represents the angle between the LiDAR beams, which has no correlation with the real distance of the LiDAR points in the world coordinate system. The front-view projections can cause multiple instances to be projected in to the same area in the feature map. It should be noted that in the 3D vehicle detection task, the vehicle is modeled with a position, size and heading angle. According to the physical constraints, the vehicle is located on the ground plane, and the pitch and roll angles are always zero. Meanwhile, the vehicles will not overlap in the bird’s-eye view, because the parallel projection discards the *z* coordinate. These characteristics are conducive to our target assignment process in Section 3.3.

#### 3.1.2. Pillar Feature Extractor

For the pillar feature extractor [5], we set pillars in the *x*,*y*-plane to group the point clouds in the detection range [0,70.4]×[−40,40]×[−3,1] on the X,Y,Z axes. If the pillar size $\left(v_{x},v_{y}\right)$ in the *x*,*y*-plane is 0.2 m × 0.2 m, all LiDAR points in the detection range are clustered into a map with a resolution of 352×400 from a bird’s-eye view. Each non-empty pillar constitutes a set of sub-point clouds $S_{x\in W,y\in H}=\left\{P_{i}\;i=1,2,...,n_{x,y}\right\}$, where each point $P_{i}$ represents a vector of (x,y,z,r), and $n_{x,y}$ is the number of points in the corresponding set. (W,H) is the range of pillar number along the X,Y axes accordingly. Since LiDAR points are non-uniformly distributed in space, the pillars contain different numbers of LiDAR points. As a result, the sub-point cloud in a pillar forms an unordered and irregular structure. We adopt a simplified version of PointNet [8] which embeds such data into a fixed-length vector by a symmetric function. For a point $P_{i}$ in a set $S_{x,y}$, we augment its vector (x,y,z) to $\left(x,y,z,r,x_{c},y_{c},z_{c},x_{p},y_{p}\right)$, just as in [5]. Let $\left(x_{c},y_{c},z_{c}\right)$ represent the component-wise distances from the current point to the arithmetic mean of all points in the pillar. $\left(x_{p},y_{p}\right)$ denote the component-wise distances between the current LiDAR point and the pillar pixel’s center to which they belong. In this manner, the entire information of a set can be defined by a tensor of size $n_{x,y}\times 9$, where $n_{x,y}$ is the point number in a sub-point cloud $S_{x,y}$. Inspired by PointNet [8], we employ a shared multi-layer preceptron with ReLU for each point in the set, resulting in a tensor of shape $n_{x,y}\times C$, where *C* is the feature channel. Then, the sub-point set feature is finally embedded into a 1×C tensor by a maximum pooling operation. Similarly, the original point cloud can be mapped to a W×H×C-dimensional tensor feature. For meshes which do not contain LiDAR points, we set 0 in all channels to make it meet the requirements.

#### 3.1.3. Parameterless Voxel Feature Extractor

According to the previous literature [3,4], a point cloud can be divided by a W×H×D voxel grid in space, along the X,Y,Z axes respectively. The traditional voxel feature extractor (VFE) [3] was implemented by a multi-layer perceptron, which contains two fully connection layers significantly increasing the difficulty of training. We apply a parameterless voxel feature extractor instead of VFE. Each non-empty voxel is represented by the arithmetic mean of its internal LiDAR points. The output of the feature extractor is a 4D tensor of size W×H×D×4. The voxel is defined as being size 5 cm × 5 cm × 10 cm. Then we apply 3D sparse convolutional blocks with a donsampling factor of *k* to extract features. The shape of the output feature map is $\left\lfloor \frac{W}{k}\right\rfloor \times \left\lfloor \frac{H}{k}\right\rfloor \times \left\lfloor \frac{D}{k}\right\rfloor \times C$. We flatten the 3D feature map to $\left\lfloor \frac{W}{k}\right\rfloor \times \left\lfloor \frac{H}{k}\right\rfloor \times \left\lfloor \frac{D}{k}\right\rfloor \cdot C$.

#### 3.1.4. Projection Relationship

From the bird’s eye view, the size of the vehicle’s footprint is invariant under changes of the heading angle. Assuming that in the x,y-plane, the detection range is $\left[x_{0},x_{1}\right]\times \left[y_{0},y_{1}\right]$ and the cell size in the feature map is $v_{x}\times v_{y}$; then the pixel located at (u,v) can be transferred to the position (x,y) in the LiDAR coordinate system, as Equation (1) shows:

(1) $$\left[\begin{array}{c} x\\ y\\ 1 \end{array}\right]=\left[\begin{array}{ccc} v_{x} & 0 & x_{0}+\frac{1}{2}v_{x}\\ 0 & v_{y} & y_{0}+\frac{1}{2}v_{y}\\ 0 & 0 & 1 \end{array}\right]\left[\begin{array}{c} u\\ v\\ 1 \end{array}\right]$$

**Figure 2:**
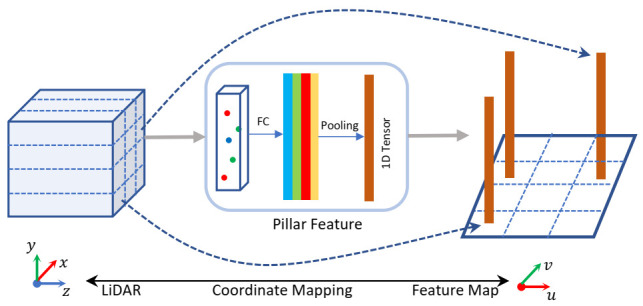
Coordinate mapping relationship between the feature map and LiDAR coordinate system, taking a pillar feature extractor as an example.

Compared with the RGB images, the pixel position of the point cloud feature map includes the spatial position prior. Figure 2 shows the correspondence between the feature map and the grid that divides the point cloud regularly in the LiDAR coordinate system. For the pillar feature extractor, the pixel size of the feature map is the size of the projection of the pillar on the X,Y-plane in the LiDAR coordinate system. For the voxel feature extractor, the pixel size of the point cloud feature map is the voxel’s size multiplied by the down-sampling factor of the convolutional layers. With the projection relationship, we map the location from the pixel coordinate system to LiDAR coordinate system easily. We designed an anchor-free detector via the feature character, which will be described in detail in Section 3.3.

### 3.2. Backbone Network

Our 3D anchor-free vehicle detector treats objects as keypoints [26] for detection. Generally, in 2D object detection task, the object occupies a large pixel region in an image, so that the 2D anchor-free detectors [7,26,35] set the down-sampling factor in the backbone networks as 32 or even larger. However, for a point cloud feature map, if the cell size is 0.2 m × 0.2 m along the X,Y axes, a vehicle object can be distributed in a circle with a radius of 8 pixels. Due to the dense arrangement of the vehicles, a large down-sampling factor may cause the target position confusion of multiple targets in the final feature map. In this work, we use a network architecture with a limited down-sampling factor similar to [3,5]. Our backbone network, as shown in Figure 1b, consists of two sub-networks: a down-sampling network and an up-sampling network. The down-sampling network is composed of a series of network blocks, which can be denoted as ConvBlock $\left(C_{in},C_{out},S_{d},N_{b}\right)$. *C* is the number of feature channels, and $S_{d}$ is the down-sampling factor of the input point cloud feature map. $N_{b}$ represents the number of convolutional layers in each block. The filter size is 3×3 in our method. We select the padding size and the step size of the first convolutional layer in each block to match the down-sampling factor $S_{d}$. For example, given $S_{d}=2$, we set the zero-padding size as 1 and the step size as 2 for the 3×3 convolution. The other convolutional layers do not change the feature map size. Note that the down-sampling factor can be 1 in the applications. The output tensor of a down-sampling block is sent to a consequent up-sampling block DeconvBlock $\left(C_{in},C_{out},S_{u}\right)$, where $S_{u}$ is the up-sampling factor of the 2D transpose convolution. Each convolutional and deconvolutional layer is equipped with a BatchNorm and a ReLU activation operation. We cascade the outputs of DeconvBlocks as the final feature map. Figure 1b shows our backbone network, in which the DeconvBlock corresponds to the up-sampling part.

Although the input feature map generated by the pillar/voxel feature extractor is sparse, it is not necessary to adopt a sub-manifold sparse convolution operation like [4] in our backbone network. Considering that the LiDAR points are distributed on the object’s surface, it is expected that there are no points falling onto the center of an object, and the element values of the corresponding feature channel are zeros. These elements are treated as non-active sites in the sub-manifold sparse convolutional operation, unable to communicate with the surrounding nodes. We apply a 1×1 convolution without bias for the detection classifier, while for the non-active sites, the response values after the convolution are all zeros. As a result, we leverage a traditional 2D convolutional layer as the backbone network, encouraging the non-active sites to communicate with their surrounding sites to aggregate efficient information.

### 3.3. Anchor-Free Detector

The ground truth for 3D object detection is defined as (x,y,z,w,l,h,θ), where (x,y,z), (w,h,l) and (θ) respectively correspond to the object’s location, dimension, and orientation. Inspired by the 2D detector CenterNet [26], we define the 3D object detection as a keypoint detection task in BEV. We get a keypoint heatmap on the X,Y-plane via a fully convolutional network, and adjust the other additional 3D information by parallel regression branches.

#### 3.3.1. Heatmap for Classification

For a 3D vehicle object detection task, given a keypoint heatmap $Y\in {\left[0,1\right]}^{W\times H}$, it is necessary to calculate the pixel center position $\left(\bar{x},\bar{y}\right)$ where the target is located. $Y_{uv}\in \left[0,1\right]$ represents the probability of a vehicle being located at the point (u,v) on the heatmap *Y*. We assume the vehicle center is at position (x,y) in the LiDAR coordinate system, the cell size is $\left(v_{x},v_{y}\right)$, the detection range on the X,Y-plane is $\left[x_{0},x_{1}\right]\times \left[y_{0},y_{1}\right]$ and the overall down-sampling factor of the backbone network is *S*. We transform the coordinate position of the vehicle center point from the LiDAR coordinate system to the heatmap coordinate system by $\left(\bar{u},\bar{v}\right)=\left(\left\lfloor \frac{x-x_{0}}{s\cdot v_{x}}\right\rfloor ,\left\lfloor \frac{y-y_{0}}{s\cdot v_{y}}\right\rfloor \right)$. Like [26], we set the Gaussian kernel function at the center point of our heatmap as the classification ground truth during training: $Y_{uv}=\exp\left(-\frac{{\left(u-\bar{u}\right)}^{2}+{\left(v-\bar{v}\right)}^{2}}{2\rho^{2}}\right)$, where ρ is the adaptive parameter reflecting the vehicle area on the heatmap [35].

**Figure 3:**
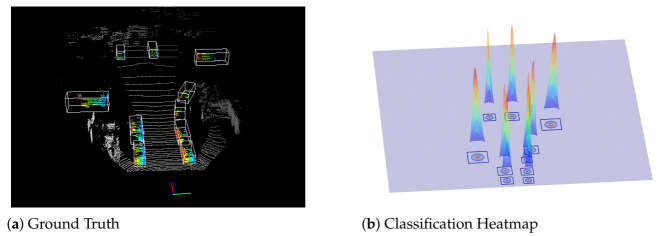
We generate classification heatmap labels (**b**) from a ground truth dataset (**a**). Labels are calculated via adaptive Gaussian distribution.

The vehicles are rigid and distributed on the ground plane; there is no overlap between vehicles from the bird’s eye view; therefore, we do not consider the overlapping of multiple Gaussian distributions for our task. Figure 3 illustrates the ground truth of a point cloud and our classification heatmap for training the detector. Unlike the anchor-based target assignment process, our method has a constant memory space occupation when generating a heatmap. Table 1 shows the comparison results. Given a training heatmap, we take the focal loss [36] for training:

(2) $$L_{heat}=-\frac{1}{N_{s}}\sum_{uv}\left\{\begin{array}{ll} \alpha {\left(1-{\hat{Y}}_{uv}\right)}^{\gamma }\log\left({\hat{Y}}_{uv}\right) & \mathrm{if}\;Y_{uv}\geq \sigma_{1}\\ \left(1-\alpha \right){\hat{Y}}_{uv}^{\gamma }\log\left(1-{\hat{Y}}_{uv}\right) & \mathrm{if}\;Y_{uv}<\sigma_{2}\\ 0 & \mathrm{otherwise} \end{array}\right.$$

We assign α=0.25 and γ=2 as the hyper-parameters of the focal loss. ${\hat{Y}}_{uv}$ is the network prediction and $Y_{uv}$ is the ground truth heatmap. $\sigma_{1}$ and $\sigma_{2}$ are the artificially set positive and negative sample cutoff values according to our soft labels. $N_{s}$ denotes the number of pixels in the keypoint heatmap satisfying $Y_{uv}\geq \sigma_{1}$ or $Y_{uv}<\sigma_{2}$. Note that $Y_{uv}\in \left(\sigma_{2},\sigma_{1}\right)$ is ignored as 0 for the classification loss. Differently from the hard labels $Y_{u,v}\in \left\{0,1\right\}$ that anchor-based methods [3,4] adopt, our method introduces non-maximum suppression in training procedure when $\sigma_{1}=\sigma_{2}=1$.

At the inference stage, we extract a set of high-value-response locations $\left\{\left(u_{i},v_{i}\right)i=0,1,\ldots ,k\right\}$ from the heatmap. Unlike [26], we apply Equation (1) to restore the center positions $\left\{\left({\hat{x}}_{i},{\hat{y}}_{i}\right)i=0,1,\ldots ,k\right\}$ of the pixels where the objects probably belong.

#### 3.3.2. 3D Information Regression

Although we obtain the pixel center $\left(\hat{x},\hat{y}\right)$ for locating the objects, $\left(\hat{x},\hat{y}\right)$ is merely an inaccurate estimation of an object’s center point. Besides, we should regress the accurate object center location (x,y,z), the object dimensions (w,l,h) and the orientation θ. These 3D parameters are regressed by multiple branches that are parallel to the classification branch. For the object center location, we regress an offset map $Y_{off}\in R^{W\times H\times 3}$. The first two channels represent the offset between an object center and the pixel center $\left(\delta_{x},\delta_{y}\right)$ in the LiDAR coordinate system. The third channel directly regresses the object location on the *Z*-axis. We use $N_{pos}$ to represent the number of the positive samples in the ground truth heatmap. The localization regression residual for each positive sample in the offset map is defined by:

(3) $$\Delta x=\delta_{x}-\hat{\delta_{x}},\;\Delta y=\delta_{y}-\hat{\delta_{y}},\;\Delta z=z-\hat{z}$$

We use the SmoothL1 loss with the same setting as [4]:

(4) $$L_{off}=\frac{1}{N_{pos}}\sum_{b\in \left(x,y,z\right)}\mathrm{SmoothL}1\left(\Delta b\right),$$

where Δb denotes the location residual. We regress a dimension map $Y_{dim}\in R^{+W\times H\times 3}$, whose channels correspond to the dimensional information (w,l,h) of the object. Since the elements in $Y_{dim}$ are always positive, the exp(·) is employed after the 1×1 convolution layer to map real numbers to (0,∞).

For the orientation angle, the anchor-based methods [3,4] regress the sinusoidal minimum error $\sin(\theta -\hat{\theta })$ between the ground truth angle and the anchor box angles. However, the sinusoidal minimum error between the angle θ and θ±π is always 0. For the methods based on sinusoidal error [4], it is necessary to apply additional direction information to correct the prediction angle. We redesign the regression labels for rotation and regress a rotation map $Y_{rot}\in R^{W\times H\times 2}$. The two channels correspond to the rotation information (cosθ,sinθ). In this way, our network is capable to decode the unique rotation angle directly. The regression residuals for the positive samples are defined by:

(5) $$\begin{array}{l} \Delta w=w-\hat{w},\;\Delta l=l-\hat{l},\;\Delta h=h-\hat{h},\\ \Delta \cos\theta =\cos\theta -\hat{\cos\theta },\\ \Delta \sin\theta =\sin\theta -\;\hat{\sin\theta } \end{array}$$

Similarly to the location offset loss function, we define dimension loss and rotation loss as:

(6) $$L_{dim}=\frac{1}{N_{pos}}\sum_{b\in (w,l,h)}\mathrm{SmoothL}1\left(\Delta b\right),$$

(7) $$L_{rot}=\frac{1}{N_{pos}}\sum_{b\in (cos\theta ,sin\theta )}\mathrm{SmoothL}1\left(\Delta b\right),$$

where Δb denotes the residuals for dimension and rotation.

#### 3.3.3. Auxiliary Loss and Joint Training

To jointly train the regression branches, we introduce the IoU layer in the detector and calculate the auxiliary loss. After decoding the bounding box (x,y,z,w,l,h,θ) of the target from the regression branches, the network applies Equation (8) to measure the 3D IoU [37]:

(8) $${IoU}_{3D}=\frac{{Area}_{\mathrm{overlap}}\times h_{\mathrm{overlap}}}{Area_{g}\times h_{g}+Area_{d}\times h_{d}-Area_{\mathrm{overlap}}\times h_{\mathrm{overlap}}}$$

The subscripts *d* and *g* denote the predicted bounding box and the ground truth respectively. The subscript overlap represents the intersection of them. Area represents the area of the bounding box projected on the X,Y-plane, whose value is related to (x,y,w,h,θ). The auxiliary 3D IoU loss is defined as:

(9) $$L_{IoU}=1-{IoU}_{3D}$$

Merely training with the 3D IoU loss leads to an acceptable AP performance. However, it cannot determine the heading angle of the vehicle. For example, the IoU between a bounding box and itself after rotating angle π is always 1. We conducted experiments and proved that only with IoU loss, the detector performs poorly on the criterion of average orientation similarity (AOS) (about 0.45). Therefore, the 3D IoU loss should be jointly adopted with other regression loss functions for training. The overall training is to optimize a multi-task loss function as Equation (10), where λ denotes the hyper-parameter scaling the loss term.

(10) $$L=\lambda_{heat}L_{heat}+\lambda_{off}L_{off}+\lambda_{dim}L_{dim}+\lambda_{rot}L_{rot}+\lambda_{IoU}L_{IoU}$$

In the inference stage, we predict $\left(\hat{x},\hat{y},\hat{z},\hat{w},\hat{l},\hat{h},\hat{\theta }\right)$ by our anchor-free detector. Given a keypoint heatmap, our network firstly extracts the pixel center position $\left(\bar{x},\bar{y}\right)$ by Equation (1) to determine where the object locates. Then it recovers the object 3D location $\left(\bar{x}+\hat{\delta x},\bar{y}+\hat{\delta y},\hat{z}\right)$ in the LiDAR coordinate system by the offset map. Meanwhile, the dimensional information $\left(\hat{w},\hat{l},\hat{h}\right)$ is inferred according to the dimension map. For rotation regression, it decodes the unique angle θ by a 2-argument arc tangent function. We screen the overlapped 3D bounding boxes with the non-maximum suppression operation.

## 4. Experiments

We evaluated the performance of our 3D detector on the KITTI dataset [38]. The KITTI training set, which contains 7481 examples, was split into a training set of 3712 samples and a validation set of 3769 samples [3]. We trained our detector for the Car class and analyzed its prediction results on three evaluation levels, i.e., the easy, moderate and hard levels. The difficulty assessment was based on the occlusion and truncation level of the objects. In the experiments, we only adopted the LiDAR point clouds within the camera’s field of view. The data augmenting the in training process was conducted with SECOND [4].

**Metrics.** We evaluated our method according to KITTI official metrics. The average precision (AP) was used to measure detection performance in KITTI dataset. AP was calculated using 11 recall sampling points in the validation set and 40 recall sampling points in the test set. The IoU threshold was 0.7 for both 3D and BEV evaluation. The average orientation similarity (AOS) was used to estimate orientation prediction performance. The AOS value ranged from 0 to 1, and 1 represents a perfect match between the orientation prediction and the ground truth.

### 4.1. Implementation Details

We set the detection range [0,70.4]×[−40,40]×[−3,1] in the X,Y,Z axes in LiDAR coordinate system, and the cell size in heatmap was 0.4 m × 0.4 m for all experiments. We tested two types of point cloud feature extractors for evaluation, i.e., the pillar feature extractor and the parameterless voxel feature extractor.

The network with pillar feature extractor is denoted as PP model, and the one with a voxel feature extractor is represented as VFE model. In PP model, we use one fully connected layer with ReLU and map input 9-channel to 64-channel output. After maximum pooling operation in each pillar, the feature extractor outputs 64-channel structured point cloud feature map. A pillar is of size 0.2 m × 0.2 m. The consecutive backbone network has three pairs of blocks: ConvBlock1(64, 64, 2, 3), ConvBlock2(64, 128, 2, 5), and ConvBlock3(128, 256, 2, 5). The outputs of the 3 blocks are up-sampled by their corresponding transpose convolutional blocks, namely, DeConvBlock1(64, 128, 1), DeConvBlock2(128, 128, 2), and DeConvBlock3(256, 128, 4). The overall down-sampling factor of the backbone network is 2. For our multi-task loss function, we set all λ=1, which means the loss function of each part has the same weight. We set $\sigma_{1}=\sigma_{2}=0.6$ for training. The network is trained for 160 epochs by a single Titan X GPU with 2 samples per batch. The Adam optimizer and one cycle learning rate are adopted. The maximum learning rate is 1.5 × 10 ^−3^, the divide factor is 10.0, and the momentum range is [0.85,0.95].

In the VFE model, the voxel size is 5 cm × 5 cm × 10 cm. After 3D sparse convolutional layers and reshape operation with down-sampling factor 8, the point cloud feature map becomes a 176 × 200 × 128 tensor. We apply three pairs of blocks: ConvBlock1(128, 64, 1, 3), ConvBlock2(64, 128, 2, 5), ConvBlock3(128, 256, 2, 5), DeconvBlock1(64, 128, 1), DeconvBolck2(128, 128, 2), DeconvBlock3(256, 128, 4). The overall down-sampling factor of the backbone is 1. We set all λ=1, $\sigma_{1}=0.8$ and $\sigma_{2}=0.4$. The network is trained for 50 epochs with 8 samples per batch. The training policy is the same with the PP model network.

In the PP model, the amount number of parameters is approximately 2.47 M. In the VFE model, the amount number of parameters is approximately 3.57 M, and about 0.99 M parameters is the 3D sparse convolution parameter. Compared with PointPillars [5], our PP model has reduced the parameter amount by about 0.08 M because of the streamlined detection head. Compared with SECOND [4], our VFE model has reduced the parameter amount by aboud 0.14 M. The inference speed of the network is related to the hardware. The official PointPillars inference time is 70 ms in our environment. For the PP model, point cloud feature extracting time is 22 ms, backbone inference time is 25 ms and the post processing time is 13 ms. For the BFE model, point cloud feature extracting time is 129 ms, backbone inference time is 41 ms and the post processing time is 18 ms.

### 4.2. Experiments on the KITTI Validation Set

We compare the proposed one-stage, anchor-free 3D detection network with other methods using the KITTI validation set. The evaluation results are shown in Table 2. PointPillars [5] did not provide an evaluation result on the KITTI validation set. Accordingly, we ran their code with the same configurations.

From Table 2, it can be found that the voxel feature extractor (in the VFE model) performs better than the pillar feature extractor (in the PP model) as our anchor-free detection head. Compared with the method of extracting features from pillars using multi-layer perceptron, the feature extractor working on small voxels with 3D convolutional layers can get more fine-grained features. For the *Cars* class, our VFE model achieved (88.31,77.97,76.17) on the easy, moderate and hard levels in terms of $AP_{3d}$, outperforming the other methods. It should be noted that our model directly generates the final prediction result from the regression branch without direction classification.

We also compare our method with other anchor-free one-stage detectors. Compared with PIXOR [10], our VFE model improved the $AP_{bev}$ by (+3.05%,+6.68%,+10.03%). The PIXOR [10] directly projected the point cloud from BEV into a 2D feature map. On the contrary, we use a more refined voxel/pillar feature extractor to process the point clouds. It should be noted that our VFE model has improved performance compared to another CenterNet-based method AFDet [24]. Unlike AFDet [24], we use a novel angle labeling method. For example, AFDet [24] regressed an 8-dimensional tensor, as it uses the 4-bin angle labeling method, while our method regresses 2-dimensional tensors for any situation. The $AP_{3d}$ of hard samples in our method is increased by +6.86% compared with AFDet [24]. Section 4.4 shows our experiment results on the regression branch. Our method is also different from CenterNet3D [25]. For example, we use an IoU loss layer by joint training and make additional improvements to the angle regression branch. Besides, we manually set the cutoff value and apply RetinaNet focal loss to deal with the imbalance of positive and negative samples. CenterNet3D added an additional corner classification branch to improve network performance, but the designing is complex. Trained by the Smooth L1 loss function, our method performs slightly better than CenterNet3D [25].

**Figure 4:**
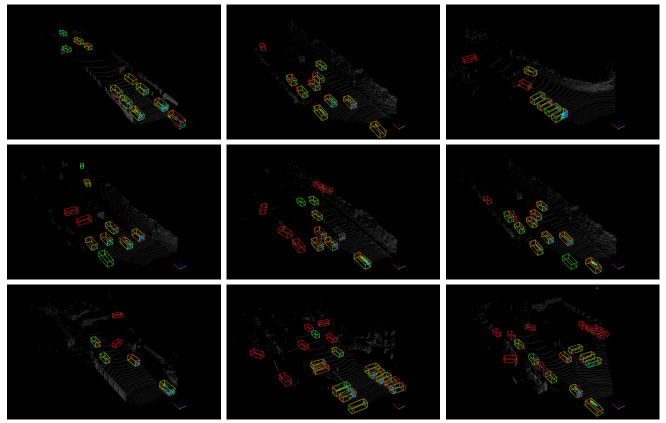
Visualization of the detection results in the KITTI validation set. The ground truth is shown in green bounding boxes. The predictions of our detector are outputs in the red bounding boxes.

Our method is at the same level of performance as the two-stage detector PI-RCNN [22] and the one-stage anchor-based detector SCNet [39]. We visualize the detect results in Figure 4. It can be observed from the visualization results that the algorithm has a high recall rate, but the false positive prediction results are also relatively high.

### 4.3. Experiments on the KITTI Test Set

There are 7518 test samples in the KITTI 3D detection benchmark. We evaluated our VFE model on the KITTI test server. Table 3 illustrates the prediction results on the KITTI test set. For the $AP_{3d}$ criterion, our VFE model reports (84.41,75.39,69.89) for the easy, moderate and hard levels, respectively. In the bird’s eye view evaluation, the VFE detection results achieved (91.58,85.83,80.54) for the three levels, respectively. Although our one-stage anchor-free network does not need any prior information about the anchor boxes during the training and prediction process, it acquires the same performance as other anchor-based, one-stage 3D detectors, such as SCNet [39], SECOND [4] and PointPainting [16].

### 4.4. Experiments on the Average Orientation Similarity

Generally, in 2D object detection task, we pay more attention to the AP evaluation. However, for 3D object detection, an accurate estimation of the orientation of the vehicle should be applied to optimize the prediction in 3D spatial domain, such as trajectory prediction, pose estimation, and so on. In this section, we apply the KITTI *validation* set to analyze the impact of 4 different regression loss function strategies on AOS estimation. The performance results are demonstrated in Table 4

We firstly use the IoU loss function for regression. After training, the network demonstrated poor AOS performance on the validation set, i.e., 44.54 on the moderate level of difficultly. Then we added a direction classification branch based on the IoU loss. The range of the vehicle orientation is divided into two intervals, and is predicted by a 0–1 classifier to correct the regression results. For example, if the regression result is not consistent with the quadrant where the classified angle is located, we correct it with a +π operation. Although this method achieves acceptable AOS results, it changes the original training label settings and adds another classification branch. The multi-bin method adds the orientation priors, identically to the anchor boxes. Moreover, it introduces more parameters to regress. We do not apply it in the fourth combined strategy. The third regression loss function is to add a regular term with a small weight constraint to the IoU loss function:

(11) $$L_{RIoU}=L_{IoU}+\lambda \sum_{a\in \left(\cos\theta ,\sin\theta \right)}\mathrm{SmoothL}1\left(\Delta a\right),$$

where we set λ=0.1 for training. The last one is the loss function shown in Equation (10), referred to as combined loss, and it performs the best.

From Table 4, it should be noted that although the RIoU did not perform badly according to the AOS indicator, its $AP_{3d}$ values, (86.87,76.91,74.81) on the easy, moderate and hard levels, were much worse than for the combined loss function.

Table 5 shows $AP_{3d}$ results of different regression loss functions on the KITTI validation set. The experimental results show that IoU loss with direction classification branch and the combined loss performed best in terms of AOS and $AP_{3d}$ indicators. It should be noted that the additional direction classification branch changes the network head’s structure. It requires additional classification loss to be designed during the training process, and increases the complexity of training label design process. Therefore, we selected the combined loss for regression, which can keep the network and training labels streamlined, and achieve an acceptable experimental effect.

### 4.5. Experiments on the Training Sample

In order to verify the impact of training sample cutoff value, we applied our detection method with different cutoff values. In these experiments, the detection range was [0,52.8]×[−32,32]×[−3,1] in the LiDAR coordinate system. The voxel size was set to 5 cm × 5 cm × 10 cm. After 3D sparse convolutional layers and the reshaping operation with down-sampling factor 8, the point cloud feature map was 132×160. The backbone contained one pair of blocks: ConvBlock(128, 128, 1, 5) and DeConvBlock(128, 128, 1). The overall down-sampling factor of the backbone was 1. We trained the network with the loss function defined in Equation (10). The training procedure contained 50 epochs, 12 samples per batch and a one-cycle training policy. We set the maximum learning rate to 0.00225, the dividing factor to 10.0 and the momentum range to [0.85,0.95]. For Equation (2), $\sigma_{1}$ and $\sigma_{2}$ denote the artificially set cutoff values of positive and negative samples, respectively. They can directly change the numbers of positive and negative samples during training.

We set $\sigma_{1}=\sigma_{2}$ as σ in Equation (2). In this case, all pixels of the heatmap are divided into two categories. From Table 6, with the cutoff value σ decreasing, the performance of our detector is significantly improved in $AP_{3d}$ and $AP_{bev}$. In the case of a high cutoff value, there are to many low-quality negative samples in the classification branch; for example, pixels around the ground-truth Gaussian peak are also marked as negative samples. In addition, since the Gaussian peak is at the center of the vehicle in *x*-*y* axis, only a few LiDAR points are distributed on the surface of the vehicle for hard samples, the pixels around the Gaussian peak should be marked as positive samples for classification. Therefore, we show additional experiments in Table 7. We filtered low-quality negative samples by setting $\sigma_{2}=0.45$, and studied the influence of the number of positive samples on the detector.

From Table 7, we find that the cutoff setting of the positive sample number has a great influence on the detection results of hard-level objects. Obviously, a smaller cutoff value $\sigma_{1}$ setting can generate more positive samples for classification and regression. The hard targets always have fewer LiDAR points on their surface, while a lower cutoff value helps improve the recall of hard samples of our detector. Table 7 shows that when the cutoff value reduces from 1.0 to 0.6, the $AP_{3d}$ value of hard samples improves by +5.75%, and the $AP_{bev}$ for hard samples improves by +6.02%.

Setting a reasonable cutoff value manually helps to improve the performance of the detector. In order to obtain better 3D detection performance, we set $\sigma_{1}=0.8$ in the final submitted detector, and a lower $\sigma_{2}$ appropriately to reduce the number of negative samples.

### 4.6. Influence of Classification Loss Function

Like the other one-stage detectors, there is an extreme imbalance problem between the positive and negative samples for classification in the 3D object detection task. For the revised SECOND-lite network with our detection head, there are about 3–10 cars in a point cloud with data augmentation, and the same number of Gaussian distributions are present during training. However, the heatmap is of size 132×160, leading to easy negative samples accumulating too much of a noisy gradient for loss function optimization. To prevent the influence of the sample imbalance problem, we compare the focal loss shown in Equation (2) (RetainNet loss [36]) and the focal loss defined in Equation (12) (CenterNet loss [35]). Table 8 shows the evaluation results for the two types of loss function.

(12) Lheat=−1N∑uv(1−Y^uv)αlog(Y^uv)ifYuv=1(1−Yuv)β(Y^uv)αotherwiselog(1−Y^uv)

α=2 and β=4 are applied for CornerNet loss function settings. We verify the effect of the two loss functions with SECOND-lite adjusted by our detection head. Like Section 4.5, there are not anchor-related parameters used in both the training and inference stages. The CenterNet loss exploits only the Gaussian peaks’ positions as positive samples. For RetinaNet loss, we set $\sigma_{1}=\sigma_{2}=0.8$ in Equation (2).

Table 8 demonstrates that, differently from CornerNet [35] and CenterNet [26], the RetainNet [36] focal loss (Equation (2)) performs better for our task. Compared with setting the negative sample weight directly, utilizing the cutoff value can generate more positive samples, which is more conducive to balancing the numbers of positive and negative samples. Finally, we apply soft labels combined with cutoff values to calculate the classification loss in our network.

### 4.7. From Anchor-Based to Anchor-Free

In 3D object detection, anchor boxes represent a set of predefined bounding boxes of a certain location, dimension and rotation. The network predicts the classification probabilities and offsets that correspond to the tiled anchor boxes. During the training process, each anchor box is assigned with a label through a similarity function. Generally, the similarity is given by two-dimensionally-rotated IoU between the anchor box and the ground truth from the bird’s eye view. For the anchor-based method SECOND-lite [4], the anchor size for vehicle detection is *w* = 1.6 m, *l* = 3.9 m, *h* = 1.56 m. Each anchor is located at z = −1.0 m with two rotations: 0 and π/2. The network output is $\left(\Delta x/d_{a},\Delta y/d_{a},\Delta z/z_{a}.w/w_{a},h/h_{a},l/l_{a},\theta -\theta_{a}\right)$ and has an auxiliary direction classification value.

**Figure 5:**
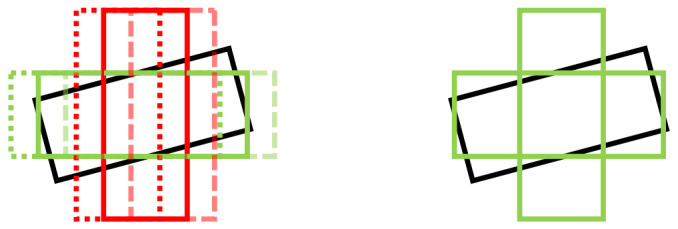
Target assigner for two rotation bin anchors from IoU labels (**left**) and Gaussian labels (**right**). Black, green and red bounding boxes correspond to ground truth, and positive and negative samples.

We firstly change the IoU-based similarity function to the Gaussian similarity function. The anchor’s *x*,*y* center is set at the center of the heatmap cell. Then each value in the ground truth heatmap can be adopted as a soft label of the anchor in the same position. We set the cutoff value as 0.6 to generate hard labels for anchors. Figure 5 demonstrates the training labels from different target assigners. The IoU-based target assigner is sensitive to the rotation of the anchor. In order to compare the effects of different similarity functions, we do not modify the training strategy of the regression branch, and retain the auxiliary direction classification branch. In this version of the network (Modified*v1* in Table 9), there are no anchor-related parameters used in the classification branch.

In order to achieve a fully anchor-free network, we modify the regression branch, and remove the auxiliary direction classification branch. We directly regress the target center *Z*-axis location and the (w,h,l) information. For the rotation regression, we exploit (cosθ,sinθ) to decode the unique rotation orientation. All of the network inference results do not correspond to the anchor-aware parameters.

Based on the model configurations described above, we apply the same Adam optimizer and training steps. For the anchor-free classification branch, we apply the loss function as Equation (2) with σ=0.6. For the anchor-free regression branch, we set all λ=2 during training.

We show the evaluation result in Table 9. Compared with the SECOND-lite, the Modifiedv1 has decreased performance across all detection indicators. Figure 5 shows the difference between Modifiedv1 and SECOND-lite in target assignment. The green anchor boxes are positive samples for classification and regression. For the Modifiedv1, we mark all anchor boxes with the same pixel positions as positive samples from Gaussian labels. The low-quality anchor boxes, such as the anchor boxes which have large differences in orientation from the ground truth bounding boxes, will be used for classification and regression branch training. This increases the difficulty of the training process, and experimental results show that there is a greater decline in the performance on hard samples. The Gaussian similarity function has an antagonistic effect on regression and classification branches based on anchor boxes.

For the Modifiedv2, we redesigned the classification and regression targets. The regression target changes from multi-bin labels to sine and cosine values. As a result, the angle prior of the anchor boxes and the additional direction classification branch can be removed. The size and position of the regression target are replaced from the anchor box residual value to the ground truth of the object. It can be found that even if the anchor-related parameters were removed, the model still performs well. The AOS results of our Modifiedv2 model is (90.50,88.29,86.91) on *easy*, *moderate* and *hard* levels respectively. The $AP_{bev}$ and $AP_{3d}$ performance is close to the anchor-based version.

## 5. Conclusions

In this paper, we introduced an anchor-free one-stage 3D detector based on LiDAR point clouds. It prevents the complex comparison procedure between the anchor boxes and ground truth, and the IoU calculation. There is no need to analyze the sample distribution of the dataset to obtain the optimal setting of a priori anchor boxes. Firstly, we generate the classification ground truth labels directly from target center locations. The regression branches predict the absolute dimensional and rotation information of the object, which is completely independent from anchor boxes. We utilize the translation matrix to convert the position from the heatmap coordinate system to the LiDAR coordinate system. Therefore, the location and size information of the massive anchor boxes will not be stored both in training and inference. Inspired by PIXOR [10], we use sine and cosine values of the ground truth to decode the unique rotation. Compared with anchor-based methods, our rotation prediction branch does not require an a priori pivot angle or an auxiliary direction classification. To deal with the imbalance of positive and negative samples, we propose the cutoff values to manually balance the ratio. We also apply the focal loss as the 2D anchor-free method CornerNet in 3D detection.To improve the performance of the regression branch, we applied IoU loss to assist in training. Finally we combined SmoothL1 and IoU loss to train the regression branch, improving both AP and AOS without any additional angle classification branch. In conclusion, we proposed a completely anchor-free method for 3D object detection, and achieved the same performance level as the anchor-based methods through a well-designed training strategy.

## Figures and Tables

**Table 1:** Tensor size comparison of our method with the anchor-based algorithms in terms of target assignment. We generated classification labels directly from the ground truth bounding boxes, which completely avoided the complicated IoU calculations using the anchor boxes and the ground truth bounding boxes. H×W denotes the heatmap size, $N_{a}$ is the number of anchors per location and *M* is the number of ground truth 3D boxes in a LiDAR point cloud.

Target Assignment	Similarity Matrix	Training Label
Anchor-based	$N_{a}\times W\times H\times M$	$N_{a}\times W\times H$
Ours	W×H	W×H

**Table 2:** Performance comparison with state-of-the-art methods. We set IoU as 0.7 for $AP_{3d}$ and $AP_{bev}$ on KITTI validation set for Car detection.

Method	Input	${\mathrm{AP}}_{3d}$			${\mathrm{AP}}_{\mathrm{bev}}$		
		Easy	Moderate	Hard	Easy	Moderate	Hard
MV3D [15]	RGB&LiDAR	71.29	62.68	56.56	86.55	78.10	76.67
AVOD [17]	RGB&LiDAR	84.41	74.44	68.65	N/A	N/A	N/A
F-PointNet [20]	RGB&LiDAR	83.76	70.91	67.47	88.16	84.02	74.44
PointPainting [16]	RGB&LiDAR	86.26	76.77	70.25	90.01	87.65	85.56
PL++ [23]	RGB&LiDAR	75.10	63.80	57.40	88.20	76.90	73.40
PI-RCNN [22]	RGB&LiDAR	88.27	78.53	77.75	N/A	N/A	N/A
PIXOR [10]	LiDAR	N/A	N/A	N/A	86.79	80.75	76.60
VoxelNet [3]	LiDAR	81.97	65.46	62.85	89.60	84.81	78.57
SECOND [4]	LiDAR	87.43	76.48	69.10	89.79	87.07	79.66
PointPillars [5]	LiDAR	86.53	77.20	70.93	89.93	87.16	85.03
SCNet [39]	LiDAR	87.83	77.77	75.97	90.35	88.09	87.30
AFDet [24]	LiDAR	85.68	75.57	69.31	89.42	85.45	80.56
CenterNet3D-SL1 [25]	LiDAR	87.92	76.84	75.74	89.97	86.81	85.85
3DSSD [40]	LiDAR	89.71	79.45	78.67	N/A	N/A	N/A
CIA-SSD [41]	LiDAR	90.04	79.81	78.80	N/A	N/A	N/A
Ours (PP)	LiDAR	82.55	75.14	72.70	89.79	86.73	84.91
Ours (VFE)	LiDAR	88.31	77.97	76.17	89.84	87.43	86.63

**Table 3:** Comparison to the state-of-the-art methods. We set IoU to 0.7 for $AP_{3d}$ and $AP_{bev}$ results on the KITTI test set for Car detection.

Method	Input	${\mathrm{AP}}_{3d}$			${\mathrm{AP}}_{\mathrm{bev}}$		
		Easy	Moderate	Hard	Easy	Moderate	Hard
MV3D [15]	RGB&LiDAR	74.97	63.63	54.00	86.62	78.93	69.80
AVOD [17]	RGB&LiDAR	76.39	66.47	60.23	89.75	84.95	78.32
F-PointNet [20]	RGB&LiDAR	82.19	69.79	60.59	91.17	84.67	74.77
PointPainting [16]	RGB&LiDAR	82.11	71.70	67.08	92.45	88.11	83.36
PL++ [23]	RGB&LiDAR	68.38	54.88	49.16	84.61	73.80	65.59
PI-RCNN [22]	RGB&LiDAR	84.37	74.82	70.03	91.44	85.81	81.00
PIXOR [10]	LiDAR	N/A	N/A	N/A	81.70	77.05	72.95
VoxelNet [3]	LiDAR	77.47	65.11	57.73	89.35	79.26	77.39
SECOND [4]	LiDAR	83.13	73.66	66.20	88.01	79.37	77.95
PointPillars [5]	LiDAR	82.58	74.31	68.99	90.07	86.56	82.81
SCNet [39]	LiDAR	83.34	73.17	67.93	90.07	86.48	81.30
CenterNet3D [25]	LiDAR	86.20	77.90	73.03	91.08	88.46	83.62
3DSSD [40]	LiDAR	88.36	79.57	74.55	92.66	89.02	85.86
CIA-SSD [41]	LiDAR	89.59	80.28	72.87	93.74	89.84	82.39
Ours (VFE)	LiDAR	84.41	75.39	69.89	91.58	85.83	80.54

**Table 4:** Average orientation similarity performance with four loss function strategies.

Loss Function	Average Orientation Similarity (%)		
	Easy	Moderate	Hard
IoU	45.23	44.54	43.97
IoU + Cls	90.59	89.02	87.83
RIoU	90.49	88.57	87.02
Combined	90.65	89.13	88.07

**Table 5:** $AP_{3d}$ with four loss function strategies on the validation set.

Loss Function	$AP_{3d}$		
	Easy	Moderate	Hard
IoU	86.31	76.88	74.98
IoU + Cls	88.18	78.15	76.82
RIoU	86.87	76.91	74.81
Combined	87.94	77.74	76.39

**Table 6:** Results for hyper-parameters. σ controls the numbers of positive and negative samples during training. When σ=1, each ground truth corresponds to the peak of the Gaussian label, i.e., only one positive sample.

Param.	${\mathrm{AP}}_{3d}$			Param.	${\mathrm{AP}}_{\mathrm{bev}}$		
	Easy	Moderate	Hard		Easy	Moderate	Hard
σ=1.0	80.69	65.36	58.37	σ=1.0	87.21	77.81	69.95
σ=0.8	81.89	72.99	66.44	σ=0.8	87.52	84.56	78.05
σ=0.6	87.45	76.75	74.44	σ=0.6	89.96	86.01	85.94

**Table 7:** Results for the number of positive samples. $\sigma_{1}$ controls the number of positive samples during training. We set $\sigma_{2}=0.45$ in this experiment.

Param.	${\mathrm{AP}}_{3d}$			Param.	${\mathrm{AP}}_{\mathrm{bev}}$		
	Easy	Moderate	Hard		Easy	Moderate	Hard
$\sigma_{1}=1.0$	86.38	75.42	68.40	$\sigma_{1}=1.0$	90.17	87.36	79.80
$\sigma_{1}=0.8$	87.14	76.68	74.84	$\sigma_{1}=0.8$	89.99	86.30	79.77
$\sigma_{1}=0.6$	86.84	76.46	74.15	$\sigma_{1}=0.6$	90.08	86.18	85.82

**Table 8:** Experiments on the loss function. For the focal loss of RetinaNet, we set the cutoff value to 0.8.

Loss Function	${\mathrm{AP}}_{3d}$			Loss Function	${\mathrm{AP}}_{\mathrm{bev}}$		
	Easy	Moderate	Hard		Easy	Moderate	Hard
RetinaNet (Equation (2))	87.17	77.05	75.66	RetinaNet (Equation (2))	89.84	87.38	86.72
CornerNet (Equation (12))	81.54	72.67	72.28	CornerNet (Equation (12))	87.92	84.67	85.19

**Table 9:** From anchor-based to anchor-free methods. SECOND-lite is an anchor-based 3D detector. Our method (v1) uses Gaussian labeling for the target assigner and keeps the same regression branch and auxiliary direction classification branch. The v2 model does not use any anchor-related parameters during training and inference.

Method	Anchor-Aware Param.		Aux. Cls.	${\mathrm{AP}}_{3d}$			${\mathrm{AP}}_{\mathrm{bev}}$		
	Cls.	Reg.		Easy	Moderate	Hard	Easy	Moderate	Hard
SECOND-lite	✓	✓	✓	88.10	77.68	75.35	90.18	87.34	86.38
Modified(v1)		✓	✓	86.84	75.98	68.63	89.88	85.71	79.35
Modified(v2)				86.84	76.46	74.15	90.08	86.18	85.82

## Data Availability

Publicly available datasets were analyzed in this study. This data can be found here: http://www.cvlibs.net/datasets/kitti/eval_3dobject.php, accessed on 9 April 2021.
